# Charting the Course Together: Municipal Top-Level Managers’ Perspectives on Fostering Safe and Integrated Care for Older Adults Living at Home

**DOI:** 10.5334/ijic.8916

**Published:** 2025-08-19

**Authors:** Heidi Hagerman, Mirjam Ekstedt, Mia von Knorring, Cecilia Fagerström, Sara Tolf, Lisa Smeds Alenius

**Affiliations:** 1 Faculty of Health and Life Sciences, Linnaeus University, Sweden; 2 Department of Learning, Informatics, Management and Ethics, LIME, Karolinska Institute, Sweden; 3 Department of Research, Region Kalmar County, Sweden

**Keywords:** complex care needs, municipal care and services, older adults, safe and integrated care, top-level managers

## Abstract

**Introduction:**

Top-level managers in municipal social care administration play a central role in ensuring high-quality care through coordination within and between organisations. However, there is limited understanding of the specific tasks and responsibilities they undertake in this regard. Therefore, this study aimed to explore municipal top-level managers’ perspectives on fostering safe and integrated care for older adults with complex care needs living at home.

**Methods:**

Thirteen top-level managers in municipal social care administration were interviewed. Interview data were analysed thematically.

**Results:**

One theme ‘Leading through trust and empowerment, and encouraging collaborations within and between organisations to foster safe and integrated care’ and five subthemes were identified: ‘Creating conditions for seamless care by minimising cross-organisational barriers’, ‘Using the mandated role when navigating the bigger picture’, ‘Empowering middle managers and nursing staff’, ‘Fostering trust in working towards a common goal’ and ‘Leveraging successful partnerships across organisations’.

**Conclusion:**

Top-level managers see themselves as parts of a larger system that requires them to collaborate with others. Empowering middle managers and nursing staff to thrive in their roles, through leadership based on trust, promotes a unified effort toward the common goal of safe and integrated care for older adults with complex care needs.

## Introduction

The increase in the number of older adults living with chronic diseases and multimorbidity [1] results in a challenge: millions of older adults worldwide with complex health and social care needs [2]. Further, the last decades of healthcare system reforms have resulted in decentralised and fragmented care [3] with fewer hospital beds and shorter hospital stays, meaning that more older adults with complex care needs are living at home [4,5]. They are often in need of coordinated care and services from a variety of healthcare and social care professionals working in different organisations [6]. However, in these decentralised care systems, care provision and coordination are challenged, as the provision of services is decided locally and may vary depending on where individuals live [3]. Thus, healthcare systems are not optimally designed to meet the needs of ageing populations, which is a globally recognised problem [6,7]. Care coordination for older adults with complex care needs is an integral part of integrated care [6] and a core task in municipal healthcare and social services [8], where top-level managers in social care administration play a substantial role [9]. There is a lack of research at the system level, focusing on top-level management perspectives on the delivery of safe and integrated care throughout the entire care pathway [10,11]. This study embraces the perspective of top-level managers’ work to foster safe and integrated care within and across organisations, as parts of a regional health- and care system.

## Background

Earlier research has concluded that healthcare organisations, to create safe and integrated care and services for older adults, must coordinate actions at multiple organisational levels and deliver integrated approaches across the entire care pathway [10,11]. A review of the literature on integrated care strategies and concepts [12] concluded that there is a need to rethink the idea of integrated care and embrace ideas of complexity. Therefore, organisational and policy efforts must coordinate work across organisational borders, allocate resources within multiple systems of funding, and promote cooperation between caregivers [6]. In recent years, there has been a collaborative policy effort between national and regional authorities, to establish a reform agenda in Sweden. The primary goal is to reshape the healthcare system by progressively shifting the focus from the hospital sector to primary care and highlighting good quality of care and person-centred services [13]. Integrated components of the reform agenda include elucidating the responsibilities of primary care and promoting enhanced collaboration between regional and municipal healthcare entities. The reform is necessary as the fragmented healthcare system faces challenges in managing patients with complex care needs and handling transitions between different providers and levels of care. The reform is also motivated by patient interests, as Swedish patients express lower satisfaction with the coordination of care than their European counterparts [5]. In addition, the number of older adults with complex care needs is growing, increasing the need for coordination between different care providers to ensure good quality care in the home environment [6].

In Sweden, the ultimate responsibility for the care and services provided within municipal operations falls on top-level managers in the social care administration. Their role involves leading and coordinating strategic planning and policy work within the administration. It also involves planning and directing daily operations as well as having responsibility for managers and subordinates in the organisation [9]. Many top-level managers’ reports having a complex mission with insufficient organisational preconditions (i.e., lack of resources and insufficient support functions) to exercise good leadership and maintain quality in their organisation. They often experience difficulties balancing strategic development, administrative work and contact with their subordinates [14]. According to a recent report from the trade union representing public service managers in Sweden, over half of the managers’ report struggling to provide the services that citizens are entitled to. Eldercare has been particularly negatively affected by financial conditions [15]. Similar challenges have been described in Norway [16]. Saving requirements, high staff turnover and a lack of competence among staff make it difficult for managers to provide safe and integrated care to older adults with complex care needs [14,15]. Previous research in municipal social care administration most often explores managers’ work on lower levels [e.g. 17,18,19] other than the top-managerial level. Although this research is of importance, it cannot be generalized to managers at all levels.

According to a study by Exley et al. [20] that studied governance and accountability for integrated care in three countries, there was a disconnection between national and regional governments resulting in blurred lines of accountability. They also found that healthcare dominated social care which undermined local collaboration. The study highlighted the local leadership as pivotal to build a common vision across organisations. According to a realist review of leaders in integrated health and social care systems [21], there is limited research on leadership in integrated care teams and systems. There is also a lack of evidence about how leaders’ approach and reason about their efforts to foster integrated care. Most of the evidence found, focuses on who the leader is, instead of what the leader does. In sum, most earlier studies have focused on leaders in general and not on the top-level managers and how they work on an overarching system level in elderly care. Given the complexities in municipal social care administration, research focusing on the system level, and exploring the top-level management perspectives on how to foster safe and integrated care is needed. Therefore, this study aimed to explore municipal top-level managers’ perspectives on fostering safe and integrated care for older adults with complex care needs living at home.

## Methods

We used an inductive qualitative approach with a descriptive design based on semi-structured interviews [22]. The consolidated criteria for reporting qualitative research (COREQ) [23] were used to obtain rigor.

### Setting and sample

In Sweden, healthcare and social care services are mainly tax-funded, and the responsibility for care services (primary care) is shared between 21 organisational regions and 290 municipalities. The regions are responsible for healthcare and rehabilitation, whereas the municipalities are responsible for social care and home healthcare. The government, at the national level, decides on legislation and financial incentives through policy aims and directives, but both regions and municipalities are largely self-governed and decide on local tax levels to accommodate local needs [5]. For municipal services, the responsibility lies with the municipal board, comprising democratically elected members from various political parties [24]. A key focus in current national policies is on supporting older adults to live independently at home [3]. In this study, we adopt the definition of older adults as individuals aged 65 years or older [25].

The participants were top-level managers in the social care administration, selected from a convenience sample of the 12 municipalities within one regional area in the south of Sweden. The area, made up of 12 municipalities with about 250,000 citizens in total, encompasses both urban areas with small and medium-sized towns and rural, sparsely populated areas. The participants were responsible for a variety of services, such as eldercare, care and daily activities for people with disabilities, municipal psychiatry, municipal healthcare and rehabilitation, and individual and family care. All top-level managers (n = 12) in the study region were contacted by e-mail, informed about the study’s aim and procedures and asked if they wanted to participate. Eleven of them accepted the invitation. One manager never replied to the invitation, despite repeated contact attempts by e-mail and telephone. One of the 11 managers who accepted the invitation was working as the temporary head of social care administration and wanted the nursing manager and the healthcare manager to participate in the interview as well. Therefore, 11 interviews were performed with a total of 13 participants, representing 11 municipalities in the region. The top-level managers had held their current positions for at least 0.5 years, overseeing a median of 15 direct subordinates, comprising mostly middle managers (range 4–26). Additionally, they had indirect responsibility for a median of 640 individuals (range 300–1,500) within the social care administration. More than half of the managers had an educational background in social work or nursing and all managers worked full-time. See Table 1 for information on manager characteristics.

**Table 1:** Characteristics of the top-level managers.


Age, years	45–64, Md 56

Years as a manager in total	4–36, Md 22

Years at this workplace	0.5–19, Md 6

Gender	

Woman	9

Man	4


Md = median.

The study reported here is part of the research project “Better care for all older people” (Kamprad Foundation no 20190249) exploring integrated care and interprofessional collaboration from a micro, meso, and macro perspective. In this study, we focused on the work of top-level managers and its potential for impact at the micro level. The research project as a whole has involved a stakeholder group comprising managers at various levels responsible for care planning, professional representatives with experience of services related to long-term conditions, and patient and family representatives with lived experiences of care. The project management group has regularly met with the stakeholder group throughout the project for planning, and to discuss and learn from findings.

### Data collection

Semi-structured interviews were conducted from November 2020 to March 2021. Due to the COVID-19 pandemic, all interviews were performed on digital platforms or by phone. The first author who performed all the interviews, is a trained qualitative researcher (PhD) with experience in studying management and leadership in eldercare. The first author had no previous relationships with the managers before the interviews. An interview guide containing three themes was created, focusing on the themes of *organisational values, own leadership and management performance* as well as *collaboration between caregivers*. The interview themes were intentionally broad to elicit reflective responses and facilitate an inductive approach. Each theme was introduced with a broad question, followed by more specific open-ended questions. Probes such as ‘Can you give me an example?’ and ‘Can you please tell me more?’ were used to acquire more information. All interviews ended with a final question asking if there was anything that the participant wanted to add that had not been said in relation to the themes. The interview guide was assessed in a pilot interview, and one question was added. The pilot interview was included in the results, as the interview guide fulfilled its aim to focus on the three themes and the interviewed participant fulfilled the inclusion criteria. The interviews lasted 50–77 minutes and were all audio-recorded.

### Data analysis

Thematic analysis [26,27] was used to inductively analyse the interview transcripts. After the interviews had been transcribed verbatim, the first author listened to the recordings and checked the transcripts, to become familiar with the text content and begin to search for meanings and patterns. Then, the text was imported into the qualitative software program NVivo v. 1.5.1 [28]. In NVivo, data extracts that were related to the study aim were coded. In the analysis, patterns in the codes were identified and sorted into initial subthemes. The process of coding and creating subthemes was performed by the first author and discussed among all the authors. Lastly, when all interviews were coded and categorised into initial subthemes, all authors discussed, reviewed, renamed and rearranged the findings in an active recursive process until consensus was reached. During this process, an overarching theme was also developed, and the final structure of the findings was set. The final forms of the subthemes and the overarching theme are presented in the results.

### Ethical considerations

The Swedish Ethical Review Authority approved the study (reg. no. 2020-01219). In line with the Declaration of Helsinki [29], all participants received written and verbal information about the study aim and procedures. They were assured of confidentiality and informed that participation was strictly voluntary, and that they could terminate participation at any time without explanation. All participants gave written and verbal consent to participate in the study. All methods were carried out in accordance with relevant guidelines and regulations [29].

## Results

The municipal top-level managers described many aspects of their approaches to fostering safe and integrated care for older adults with complex care needs. They described how they were leading through trust and empowerment and how they encouraged collaborations both within and between organisations. They also discussed the importance of creating conditions for seamless care for older adults living at home by minimising cross-organizational barriers. The informants stated that they used their mandated role when navigating the bigger picture of preconditions and challenges and that it helped them gain a sense of control over their work. To create safe and integrated care for older adults, it was essential to empower middle managers and nursing staff to excel in their roles. By exerting leadership based on trust, the top-level managers tried to make all the individuals in the organisation work toward a common goal. However, even though the managers were at the top-level in their respective organisations, they were also part of a larger system. This required them to leverage successful partnerships both within and between organisations, to meet the increasing demands for care of older adults with complex care needs. Table 2 presents an overview of the subthemes and the overarching theme.

**Table 2:** Overview of subthemes and overarching theme.


SUBTHEMES	THEME

Creating conditions for seamless care by minimising cross-organisational barriers	Leading through trust and empowerment, and encouraging collaborations within and between organisations to foster safe and integrated care

Using the mandated role when navigating the bigger picture	

Empowering middle managers and nursing staff	

Fostering trust in working towards a common goal	

Leveraging successful partnerships across organisations	


### Creating conditions for seamless care by minimising cross-organisational barriers

Many top-level managers stated that the core value of their organisation was to prioritise a preventive and health-promoting approach to their mission, so that older adults could stay independent in everyday life for as long as possible. However, the informants described that achieving equitable, safe, and integrated care for those in need of assistance required middle managers and nursing staff to meet the individual needs of each older adult. This could be accomplished by providing care based on the preference of the older adult, within the comfort of their own home. The top-level managers emphasised that delivery of high-quality care required continuity in care and that they created the necessary conditions for their middle managers and nursing staff to collaborate closely with other healthcare professionals in the region. In a successful collaboration, the organisational borders between the different care providers should not create any worry/concern for the older adult. This was described as follows:

‘I feel like keeping track of borders doesn’t have to mean arguing with each other. If they are clear, I think that you can collaborate well, because then you know where the borders are and you also know when you sometimes have to step over the fence a bit and when to step back, because then you can patch things up sometimes.’ (Interview no 4)

All informants stated that it was important that they had an interpersonal perspective and were dedicated to the work with older adults. The managers described that their humanistic and democratic motive force meant that they were able to realise changes for those in need and have an impact on the eldercare organisation.

‘The cliché is this stuff about making a difference, but it is actually about … yeah, it is really about that, being able … to contribute to making people’s everyday life a bit easier.’ (Interview no 9)

However, the core values for organisational work were described as multifaceted. Some top-level managers said that the organisational core values were not always visible or in the foreground in their everyday work, whereas others said that the core values corresponded to their own values and therefore were an integral part of their work. Most study informants described having the goal that all individuals working in their organisations would treat the older adults with empathy and respect to create a sense of safety and comfort among them.

### Using the mandated role when navigating the bigger picture

Most informants said that they had a broad decision-making mandate in their role, as long as their actions remained within the prescribed boundaries of legislation, budgets, policies and political vision. They stated that their superiors and politicians placed their trust in them, granting them significant autonomy to manage their work independently.

‘I have a lot of … I think most managers in social care do, a broad mandate over their own things, their operation, their budget and so on, and expectations to match, of course.’ (Interview no 3)

The executive management team was described as essential for the top-level managers to manage all the different parts of their responsibilities. To ensure high-quality care that fostered safety and security for older adults, as well as to address strategic challenges for the future, the informants and their executive teams integrated feedback from surveys, national quality registers and forums in response to requests from citizens and nursing staff.

‘Now I need to start thinking about what things might look like in four or five years in [name of their municipality] and where I think we’re headed – what should it look like here?’ (Interview no 8)

The top-level managers described using multiple communication channels to enhance communication within the organisation and ensure the engagement of all their middle managers and nursing staff. This included regular meetings and ‘theme days’ with superiors, politicians, middle managers and citizens, alongside written information channels such as e-mails, newsletters, intranet and information screens. However, as noted by some study informants, achieving this level of communication required digitalisation, something not yet realised in all organisations.

Another important part of the managerial role that the informants highlighted was to cooperate with others and encourage middle managers and nursing staff to collaborate with other professionals at all levels. This included both other departments in their own municipality, and other municipalities, as well as other caregivers and organisations. The informants were cognisant of the evolving demands for cross-organisational collaboration during the transition to providing care closer to the patients, and they openly expressed their concerns.

‘We’re moving toward even more people being cared for at home and more person-centred care and that means that we … we and the healthcare centres need to be in lockstep for that to work.’ (Interview no 11)

This collaboration was usually described as well-functioning. However, one large challenge that the informants pointed out was how to stay within budget when the region shifted the responsibility of care to the municipal organisation. They also described sociodemographic challenges, challenges with nursing staff lacking sufficient skills in the Swedish language, and challenges in recruiting, educating and retaining competent caregiving staff, all potentially affecting the quality of care.

‘Our challenges // will be ensuring competence provision, that it … that we can keep it at such a level that we have quality in all our operations.’ (Interview no 11)

Some informants, working in smaller municipalities highlighted that this could be advantageous, in terms of working closely with key persons and gaining insight into the quality of care in their organisation. Furthermore, decision-making pathways were short.

‘I think we have pretty good general knowledge. I find out a lot. I get a good overview and that means that we … we can quickly adjust if we feel that something is going wrong. // if we get deviation reports or input, I’ll find out about it. The decision-making pathways are shorter. I meet people and have an insight into the operations.’ (Interview no 8)

On the other hand, working in a smaller municipality was also described as challenging. One challenge described was the limited administrative support in the organisations, resulting in a few individuals having to do a large variety of different tasks. This made it hard to obtain specialist competence in some areas. A few informants stated that it was essential that small municipalities cooperate in regarding specialist competence, to create safe and integrated care that could provide for all the older adults’ needs. Another challenge described was when municipalities received targeted government grants focusing on competence development among nursing staff. Such grants often need to be used within a short timeframe and some managers had a hard time finding substitutes who could free up time for their nursing staff to attend training.

### Empowering middle managers and nursing staff

Many informants said that it was important that they allocated resources (i.e., budget, staff, facilities, equipment, and competence development) to their middle managers to provide them with the necessary prerequisites to establish safe and integrated care for older adults living at home. The informants also described their desire for middle managers and nursing staff to be able to develop within their roles, encouraging them to take ownership of their work and explore new ideas. They noted that this often resulted in increased self-confidence and contributed to organisational development. In order to help their subordinates try out new ideas, the top-level managers tried to create the necessary preconditions and support their coworkers even if an idea failed. Many study informants also highlighted that it was their responsibility to create the conditions for their nursing staff to work in multi-professional teams in the care of older adults.

‘Like the multi-professional team, as a strength, as a success factor, specifically to provide holistic … good care to the care recipients. I think that’s something you should want to highlight.’ (Interview no 6)

### Fostering trust in working towards a common goal

The informants described that most of the leadership that they exercised was at a strategic level, and they exercised indirect leadership within their organisations. One manager said:

‘If I think about my personal leadership, that’s related to my closest middle managers, because I lead through them, by giving them space, so that I’m not micromanaging every aspect … to make them feel insecure or unsure about what their responsibilities and mandates are, but rather that they feel they have space and get good support from me.’ (Interview no 4)

Although the informants exerted indirect leadership in their work for the older adults, it was important that they made themselves available and always supported the middle managers. An essential aspect mentioned by the informants was creating a safe and trusting work environment where middle managers had sufficient degrees of freedom to adapt their work in line with their own intuition and the wishes and needs of the older adults. Some informants mentioned that it was important to create preconditions and a clear framework, so the middle managers and nursing staff could be involved in their work and confidently take on responsibilities. This was considered to enable a person-centred approach based on the older adults’ requests and needs and counteract the tendency toward overly standardised care.

‘I think of it like this, in order for you as an employee to do a good job and provide security to a citizen, you as an employee also need to feel a sense of security and understand like the framework and like … the structure and everything. If you don’t feel that as an employee, I think that however hard you try, the citizen is likely to pick up on that too.’ (Interview no 1)

The informants emphasised their role in ensuring that all individuals across the organisation moved in synch. To achieve this, active listening, dialogues and effective communication were essential. By establishing a shared understanding, with explicit goals in the work for older adults, the managers encouraged collaboration between team members to achieve these goals.

‘That’s to ensure that our employees are involved in the work on Person-centred and Integrated Care [an ongoing national reform]. Everyone from the operative manager to the unit manager and on to the licensed vocational nurses at the very end. That we’re clear on what we want.’ (Interview no 6)

Some informants described that they had participated in various leadership programmes and thereby gained a shared understanding and language that helped them communicate and motivate others within their organisations. Many study informants mentioned the importance of supporting middle managers to develop their own individual leadership styles, so they would have the best prerequisites to exercise leadership in their work with older adults. One informant stated that to be a successful leader, one should have a non-prestigious approach to the role. This meant being transparent, trying out new ideas, and admitting mistakes, without seeing them as failures.

Other kinds of support that were described included operatively helping middle managers with difficult issues at their units. This involved acting as a sounding board for them and dealing with issues which were not directly related to the daily operations, to enable the middle managers to focus on the core aspects of their work.

‘Sometimes I feel like it’s my responsibility to kind of provide an umbrella so that the rain won’t make its way in … it can rain on me, but not on my employees, they shouldn’t have to … It’s all about protecting them sometimes if … if certain things involve various duties and that kind of thing … “you do your work and we’ll make sure to figure this out together somehow” // sometimes it can be the government and sometimes it can be newspapers and, yeah, it varies, who I need to shield them from with the umbrella.’ (Interview no 2)

However, although the informants most often described situations when the middle managers’ leadership worked well, they sometimes had middle managers who did not fulfil their role in a successful manner. The informants had to act in such situations – even though this was considered difficult – as it would otherwise negatively affect the care provided to older adults.

### Leveraging successful partnerships across organisations

The informants described collaboration with other care service providers as essential to their work and their organisations, to address future challenges. Being a small municipality was considered to facilitate collaboration with others, as only a few care providers were involved in older adults’ care. Collaboration with local healthcare centres was considered particularly important when targeting older adults. Although such collaborations were often described as well-functioning, many informants described having regular meetings with managers at local healthcare centres to develop the collaborations further. At these meetings, they could discuss any potential issues or developments, as well as strategies to meet the older adults’ needs, and thereby reduce the risk of the organisations growing isolated from each other.

‘I think we all see the need for each other, and it benefits the people we are supposed to help if we collaborate, so no one is territorial or anything like that – that if we can help each other, we will.’ (Interview no 1)

The managers also described having monthly meetings with other municipal top-level managers in the social care administration and having regular meetings with managers working in the region. The purpose of these meetings was to come together and discuss strategic issues, shared goals and commitments so that all caregivers could maintain a unified approach to providing safe and integrated care for older adults. The informants pointed out that successful collaborations hinged on having a non-prestigious approach, showing courage and genuinely wanting to collaborate with others. Many managers highlighted personal relationships as success factors for collaborations. However, as collaborations could be negatively affected by employee turnover, it was particularly important for the informants to build sustainable organisational structures for collaboration with other care organisations. As one informant put it:

‘It shouldn’t be too closely tied to any one individual. I think it should be resilient even if large numbers of people are swapped for other people, simply because we have built a sound structure.’ (Interview no 6)

Another factor that facilitated collaboration between care organisations was the use of shared digital solutions. Although the informants were optimistic about collaboration across care organisations, a lack of resources or inherent power structures sometimes hindered collaboration. For example, although the organisations had common goals of collaboration, differing organisational cultures, differing preconditions and differing approaches to achieving the goals could decrease the possibilities for collaboration.

‘There are twelve municipalities in the region, and we’re supposed to work in the same direction and do that together with the region, which is a very large organisation … And then when you meet at the local level, like unit managers, nurses and the local healthcare centre, then you’re all supposed to feel that you’re working from the same platform, which is a challenge.’ (Interview no 4)

Another barrier that complicated collaboration was related to finances and short-term decisions in the organisations. As care pathways often run through multiple different departments and organisations, financial cutbacks in one organisation could have ripple effects on the others. The need for a holistic understanding of the bigger picture to ensure coordinated and effective decision-making was emphasised. As one informant said:

‘If you really want to collaborate and cooperate, you need to talk about the broader strokes, not just about my little building block in the context.’ (Interview no 9)

## Discussion

This study revealed that although municipal top-level managers had the autonomy to manage their work and exercise leadership based on trust, to empower everyone in the organisation to work toward a common goal, they also recognised that each organisation was part of a larger system. This necessitated collaboration with others, both within and between organisations, to meet the increasing demands for care and to foster safe and integrated care for older adults with complex care needs. This study offers insights into how top-level managers in the social care administration foster safe and integrated care within and across organisations, as part of a regional health and care system. Our results are in line with the conceptual values for integrated health services delivery (i.e. the personal (e.g. ‘trustful’), the professional (e.g. ‘collaborative’), the management (e.g. ‘efficient’), and the system level (e.g. ‘comprehensive’)) as described by Zonneveld et al. [30].

Responding to calls in previous research [10,11,31], this study emphasises the role of the larger system – rather than the isolated parts – in improving the integration of care. This is important considering the lack of evidence on leadership in integrated health and social care systems, especially on leaders on the system level [21]. The results of this study showed that many of the informants embraced the complex system perspective and encouraged middle managers to create their own understanding of what needed to be done within their specified boundaries, to enable collaboration across the system. Eldercare can be characterised as a complex system [32], and as such, the system contains multiple interactions between people, tasks, technology, organisational structures and external factors [33]. When isolated parts of a system interact with other parts, this might trigger unintended and unpredictable reactions [34], and integration at one point can lead to fragmentation elsewhere [35]. For example, this study showed that financial cutbacks in one organisation could have a ripple effect on other municipal organisations. In line with findings from Svensson et al. [36], the informants in this study argued to be able to deal with such chain reactions, individuals need to be prepared and ready to act in case of unpredictable events. Further, McNab et al. [33] argue that individuals need to be able to look beyond the individual components and instead act upon what emerges, due to the unpredictable interactions between different system components. Our findings thus corroborate Hughes et al. [12] conclusion, that there is a need to rethink the idea of integrated care. Instead of attempting to find an ideal, or unified experience, practice and theory, it is important to embrace ideas of complexity to understand integrated care as dynamic and inseparable from the context.

Most of the top-level managers in this study stated that they had a broad scope of authority in their role, and they experienced that their superiors and politicians placed trust in them, granting them significant autonomy to manage their work independently, which is similar to the findings in Hagerman et al. [17]. This is relevant to highlight because access to supportive structures (i.e., structural empowerment), as outlined by Kanter [37], fosters positive workplace outcomes for both managers and subordinate staff. A previous study on managers in eldercare [38] argued that access to structural empowerment leads to higher self-reported leadership and management performance. Many informants in this study stated that it was important for them to empower middle managers and allocate the resources necessary to enable middle managers to work toward safe and integrated care within their organisations. This is in line with previous research [37,39] stating that managers with high access to empowerment can motivate and empower their subordinate staff. It also positively affects the subordinate staff’s ratings of their managers’ leadership and management performance [39]. This is crucial to understand, as many top-level managers describe facing a challenging work situation within eldercare, with limited time for their subordinate staff [14].

In this study, the top-level managers described encouraging collaboration among team members to establish a common understanding with explicit goals in their work for older adults. This is in line with the point that Swanson et al. [31] make about the importance of embracing collaborations across disciplines and organisations through iterative dialogues, to develop a shared vision among diverse stakeholders and to develop systems thinking in healthcare. In this study, the informants described various strategies for building networks and organising regular meetings with other municipal and regional top-level managers to discuss and come to an agreement on common goals and arrangements to ensure that all caregivers provided safe and integrated care to older adults. This approach mirrors findings in a study by Svensson et al. [36], exploring the perspectives of top-level managers in healthcare in the same region. They show how the managers worked to create collaborative networks to identify critical gaps between different organizations and stakeholders to ensure seamless care for all inhabitants [36]. The value of frequent meetings and communicating effectively between stakeholders to focus on key objectives and keeping a shared vision has also been highlighted in a systematic review by Iflaifel et al., [40], as key factors to developing resilient and integrated health care.

Many top-level managers in this study described focusing on strategic leadership and operating through indirect leadership within their organisations. They placed trust in their middle managers and emphasised the importance of being accessible and supporting the middle managers to foster autonomy and help others cultivate their own unique and effective leadership styles. Like our findings, Hasche et al. [41] suggest trust is intertwined in both vertical and horizontal relationships. Specifically, high trust between managers and subordinates can positively impact trust among peers at both the managerial and the subordinate levels [41]. The informants also described that they encouraged middle managers to try out new ideas and admit mistakes without seeing them as failures. These leadership behaviours were designed to foster a culture of tolerance, enabling employees to feel safe in raising organisational issues. Similar results have been reported by Ree et al. [42], stressing the importance of learning from errors and to encouraging employees to find solutions to mistakes together. The informants in this study expressed a commitment to shielding middle managers and assuming responsibility for any consequences, if necessary. Studies [see e.g. 43] have reported positive reactions among followers when leaders show trusting behaviour. These reactions include increased motivation, job satisfaction and self-confidence, improved efficiency, and a sense of appreciation for and closeness to the leader. However, it is essential to acknowledge that followers have also reported negative reactions, such as working more hours, feeling abandoned, and experiencing pressure due to leaders’ trusting behaviour [43]. The current study focused on top-level managers’ perspectives on building trust through their leadership. However, it is important to be aware of the potential negative experiences of followers, even when the intention behind building trust is positive.

### Methodological considerations

The top-level managers interviewed in this study had varied experiences and background characteristics, which adds to the credibility of the findings. By using a convenience sample including 13 top-level managers representing nearly all municipalities in one regional area in the south of Sweden, the transferability of findings might be limited. This sampling method may introduce selection bias, as the chosen municipalities may not be representative of the entire population. However, a comprehensive presentation of the sample, method and analysis will enable readers to assess the transferability of the findings to other contexts. In the results section, verbatim quotations from the interviews have been used to illustrate analytical points and to enable readers to verify findings against the raw data. In the data analysis in NVivo, reflexive memos were written during the coding process, and all researchers were engaged in an iterative data analysis process, which promoted dependability [27,44].

## Conclusion

Our findings highlighted the important role of top-level managers in facilitating and encouraging collaborations within and between organisations to foster safe and integrated care for older adults. Although the informants described high degrees of freedom to exercise leadership based on trust and how they worked to empower the individuals within their respective organisations, they were also aware of being a part of a larger system that required collaboration with others. The top-level managers in this study strove to provide multiple arenas for collaboration at all levels, both horizontal and vertical, within and between organisations and across different systems. They hoped to thereby ensure an empowered and collaborative environment to support their staff in the provision of safe and integrated high-quality care for older adults with complex care needs living at home.

## Data Accessibility Statement

The interview data that were collected and analysed in this manuscript are not publicly available due to participants not having consented to public availability. Aggregated data in Swedish are available from the corresponding author upon reasonable request.
